# Evaluation of cerebral hemodynamics by transcranial Doppler ultrasonography and its correlation with intracranial pressure in an animal model of intracranial hypertension

**DOI:** 10.1590/0004-282X-ANP-2020-0591

**Published:** 2022-02-21

**Authors:** Matheus Schmidt SOARES, Almir Ferreira de ANDRADE, Sérgio BRASIL, Marcelo DE-LIMA-OLIVEIRA, Alessandro Rodrigo BELON, Edson BOR-SENG-SHU, Ricardo de Carvalho NOGUEIRA, Daniel Agustin GODOY, Wellingson Silva PAIVA

**Affiliations:** 1 Universidade de São Paulo, Faculdade de Medicina, Departamento de Neurologia, São Paulo SP, Brazil.; 2 Hospital Carlos G. Malbrán, Sanatorio Pasteur, Unidad de Cuidados Intensivos, Catamarca, Argentina.

**Keywords:** Intracranial Pressure, Intracranial Hypertension, Ultrasonography, Doppler, Transcranial, Models, Animal, Pressão Intracraniana, Hipertensão Intracraniana, Ultrassonografia Doppler Transcraniana, Modelos Animais

## Abstract

**Background:**

Transcranial Doppler has been tested in the evaluation of cerebral hemodynamics as a non-invasive assessment of intracranial pressure (ICP), but there is controversy in the literature about its actual benefit and usefulness in this situation.

**Objective:**

To investigate cerebral blood flow assessed by Doppler technique and correlate with the variations of the ICP in the acute phase of intracranial hypertension in an animal model.

**Methods:**

An experimental animal model of intracranial hypertension was used. The experiment consisted of two groups of animals in which intracranial balloons were implanted and inflated with 4 mL (A) and 7 mL (B) for controlled simulation of different volumes of hematoma. The values of ICP and Doppler parameters (systolic [FVs], diastolic [FVd], and mean [FVm] cerebral blood flow velocities and pulsatility index [PI]) were collected during the entire procedure (before and during hematoma simulations and venous hypertonic saline infusion intervention). Comparisons between Doppler parameters and ICP monitoring were performed.

**Results:**

Twenty pigs were studied, 10 in group A and 10 in group B. A significant correlation between PI and ICP was obtained, especially shortly after abrupt elevation of ICP. There was no correlation between ICP and FVs, FVd or FVm separately. There was also no significant change in ICP after intravenous infusion of hypertonic saline solution.

**Conclusions:**

These results demonstrate the potential of PI as a parameter for the evaluation of patients with suspected ICP elevation.

## RESUMO

**Antecedentes:**

O Doppler transcraniano (DTC) é uma técnica não invasiva para a avaliação da hemodinâmica cerebral, porém existem controvérsias na literatura sobre sua aplicabilidade preditiva em situações de elevada pressão intracraniana (PIC).

**Objetivo:**

Investigar o fluxo sanguíneo cerebral pelo DTC e correlacioná-lo com as variações da PIC na fase aguda da hipertensão intracraniana em modelo animal.

**Métodos:**

Dois grupos de animais (suínos) foram submetidos a hipertensão intracraniana secundária à indução de diferentes volumes de hematoma, por meio da insuflação de balão intracraniano controlado com 4 e 7 mL de solução salina fisiológica (grupos A e B, respectivamente). Em seguida, administrou-se infusão venosa de solução salina hipertônica (SSH 3%). Foram coletados os valores dos parâmetros de PIC e DTC (velocidade sistólica [FVs], diastólica [FVd] e média [FVm] do fluxo sanguíneo cerebral), bem como o índice de pulsatilidade (IP). Comparações entre os parâmetros do DTC e o monitoramento da PIC foram realizadas.

**Resultados:**

Vinte porcos foram estudados, dez no grupo A e dez no grupo B. Correlação significativa entre IP e PIC foi obtida, principalmente logo após a elevação abrupta da PIC. Não houve correlação entre PIC e FVs, FVd ou FVm separadamente. Também não houve alteração significativa na PIC após a infusão de SSH.

**Conclusões:**

Esses resultados demonstram o potencial do IP como um bom parâmetro para a avaliação de pacientes com suspeita de elevação da PIC.

## INTRODUCTION

Intracranial hypertension (ICH) is a challenging clinical condition in the management of patients with acute intracranial lesions. Many conditions can lead to an abrupt increase in intracranial pressure (ICP), such as traumatic brain injury (TBI), stroke, spontaneous intracerebral hemorrhage (SICH), hydrocephalus, infections, brain tumors, etc^1^ ^,^ ^2^ ^,^ ^3^ ^,^ ^4^.

Invasive ICP monitoring is an important adjunct in the clinical management of ICH, although there are no studies with strong evidence of its benefits^5^. In a trial published in 2012, invasive ICP monitoring was not associated with better patient outcome compared to clinical and tomographic evaluations^6^. Nonetheless, this technique remains the gold standard method for ICP assessment according to the current Brain Trauma Foundation (BTF) guidelines^5^.

With this in mind, techniques based on transcranial Doppler (TCD), a non-invasive method easily accessible at bedside, have been studied. Elevation of ICP leads to changes in the cerebral wave pattern and blood flow velocities obtained by TCD^7^ ^,^ ^8^ ^,^ ^9^.

While TCD is a promising method for bedside evaluation, studies that investigated it as a surrogate of ICP have controversial results, especially because of influences of systemic factors on flow velocities and debate concerning pulsatility index (PI) as an indicator of whether ICP or cerebral perfusion pressure. Further studies are needed to confirm this hypothesis^10^ ^,^ ^11^ ^,^ ^12^. Thus, in this experimental study, we aimed to correlate cerebral blood flow assessed using Doppler technique with the variations of the ICP in acute phase of intracranial hypertension in an animal model.

## METHODS

This experimental study was previously approved by the Ethics Committee for Research Projects of the University of São Paulo Medical School. All applicable institutional and national guidelines for the care and use of animals were followed.

### Animals

Hybrid pigs of the Landrace, Duroc, and Pietrain breeds were used. These were brought into the laboratory on the day of the experiment.

### Anesthesia protocol

The animals were pre-anesthetized with ketamine (Ketamin-S^®^, Cristália) at a dose of 5 mg/kg and midazolam (Dormire^®^, Cristália) at a dose of 0.25 mg/kg, both placed in the same syringe and administered intramuscularly. These drugs were selected because they have no significant influence on ICP and cerebral blood flow^13^ ^,^ ^14^. After 15 minutes, the marginal ear vein was punctured with a 20- or 22-gauge vascular catheter (BD Insyte^®^). Intravenous anesthetic induction with propofol (Provine^®^ 1% - Cláris) was performed at a dose of 5 mg/kg. The animals were submitted to orotracheal intubation with an endotracheal tube of 6 mm diameter (Portex^®^), and anesthetic maintenance was performed with propofol (Provine^®^ 1% - Cláris) at a dose of 3 mg/kg/h and analgesia was maintained with fentanyl (Fentanest^®^ - Cristália) at an initial dose of 5 μg/kg followed by continuous intravenous (IV) infusion of 0.4 μg/kg/min. Neuromuscular blockade was obtained with pancuronium (Pancuron^®^, Cristalia) bolus at 0.1 mg/kg IV followed by continuous infusion of this agent at a dose of 0.02 mg/kg/h.

After intubation, the animals were submitted to volume-controlled mechanical ventilation (Dixtal^®^ 5010 Ventilator). Through an abdominal medial incision, cystostomy was performed under direct vision to control diuresis of the animal. The right femoral artery was punctured and connected to a pressure transducer in all animals for monitoring of invasive mean arterial pressure. Arterial blood gas analysis was performed with samples of 0.3 mL at the beginning of the procedure (in order to establish ventilatory parameters), and after interventions, to evaluate maintenance of the physiological parameters.

### Experimental procedure

The ICH animal model developed and previously validated by this research group was used in the present study^15^, inclusive for TCD assessment in swine^16^ ^,^ ^17^. The model simulates a right frontal intracerebral hemorrhage, performed in a controlled manner. An L-shaped fronto-temporal incision was performed on the head of each animal, at the midline and temporal region just in front of the ear to expose the coronary and sagittal sutures. Then, a bone trepanation 1 cm lateral to the sagittal suture and 1 cm anterior to the coronal suture was made in the right hemicranium, through which the intraparenchymal catheter (Neurovent-P^®^, Raumedic^®^, Munchberg, Germany) was inserted for invasive ICP monitoring in the frontal lobe white matter. A bone trepanation located 1 cm lateral to the sagittal suture and 1 cm posterior to the coronary suture allowed the introduction of an 8-French pediatric vesical catheter, reaching the subcortical white matter. Then, infusion of 0.9% NaCl solution (PS) was performed for 15 minutes, controlled with infusion pump (Infusomat^®^ compact, B Braun^®^, Melsungen, Germany). A small ipsilateral temporal trepanation was also performed to allow the accomplishment of cerebral Doppler ultrasound with a 5-8 MHz transducer (SonoSite - Micromax, FUJIFILM SonoSite, Washington, DC, United States). This allowed to analyze the cerebral blood flow velocity, establishing the systolic blood flow velocity (FVs), the diastolic velocity (FVd), and from these, the derived parameters were obtained: mean blood flow velocity (FVm=FVs+2xFVd/3) and the pulsatility index (PI) (FVs-FVd/FVm).

The animals were divided into two groups (A and B), in which intracranial hypertension was induced by the inflation of the intraparenchymal balloon with two different volumes, as described below (Table 1). Normal parameters were calibrated in both groups in the first hour.

**Table 1.** Experiment time points.

Group	0h to 1 h	1h from start	2.5h from start	3h from start	4h from start
A	Settings	4 mL balloon inflation	3% HS infusion	Balloon deflation	End
B	Settings	7 mL balloon inflation	3% HS infusion	Balloon deflation	End

HS: hypertonic saline solution; h: hour.

In group A, the balloon already implanted in the frontal white matter was infused with 4 mL of PS, and soon after, the multiparametric data were collected, which included ICP and the evaluation by TCD. This hematoma is equivalent to an expansion of approximately 80 ml in a human adult brain. This equation is based on the proportion of the brain weight of the animal of 2 months and 18 kg (average of 75 g) relative to normal adult brain weight (1500 g), with a 5% relation. In group B, a 7 mL infusion of PS was performed, equivalent to a volume of approximately 140 mL in a human adult brain^15^.

In both groups, one hour and 30 minutes after onset of balloon inflation, intravenous infusion of hypertonic saline solution (HS; 3% NaCl solution at the dose of 5.3 mL/kg) was performed. After another 30 minutes, we proceeded with balloon deflation, corresponding to the simulation of a surgical procedure.

During the experiment, several parameters were monitored including clinical parameters (pupils), invasive mean arterial pressure (MAP), parenchymal ICP, and TCD measurements (FVs, FVd, FVm, PI) obtained bilaterally from the middle cerebral arteries. These data were collected before and after all interventions on the animals.

At the end of each experiment, the animals were sacrificed through an intravenous dose of propofol (20 mg/kg) and fentanyl (10 mg/kg), followed by 40 mL of 19.1% potassium chloride solution. After the sacrifice, the animals were placed in plastic bags, with labels that clearly identified the origin, content, and the responsible researcher. They were then transported to be incinerated according to our institution routine protocol.

### Statistical analysis

Statistical analysis was presented through means and standard deviations, as well as graphs of individual and medium profiles. For each of the measurements, including ICP, adjusted linear mixed regression analysis were applied, considering random effect in the intercept and normal distribution for the random effects^18^. Spearman correlation was calculated for PI and ICP values. The graphical analysis indicated that the random effect of the intercept appeared to differ between groups as well (experiment effect variability in group B was higher than in group A). Therefore, besides considering a random effect of the individual, the effect was considered distinct between groups. The analyses were performed using the R 3.4.0 software (R Core Team, 2017, Vienna, Austria). The results were interpreted using a significance level of 5%.

### RESULTS

Twenty two-month-old hybrid pigs with an average weight of 18.46 kg (±1.12) were studied. They were divided in two groups of ten animals: group A (4 males, 6 females) and group B (4 males, 6 females). One pig in group B died before the end of the experiment and was excluded from the analysis. All animals were hemodynamically stable during the experimental procedure, except two animals of group B that presented refractory low blood pressure.

**Figure 1.**
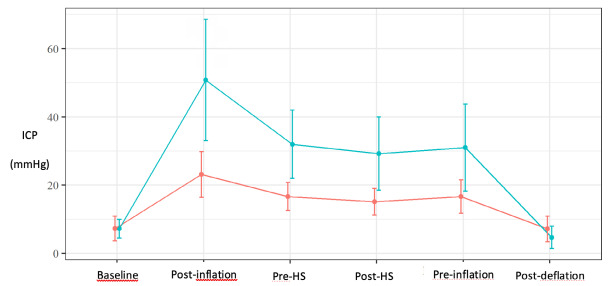
Average profile of intracranial pressure per group.

Table 2 shows the means and standard deviations observed for ICP measurements collected from the intraparenchymal monitoring and the TCD-based variables FVs, FVd, FVm, and PI. A moderate elevation of ICP was observed in group A and a significant increase was observed in group B after inflation of the balloon (Figure 1). No major ICP variation between the pre-HS and pre-deflation moments were observed in both groups.

**Table 2.** Means and standard deviations of measurements by group and time point.

Parameter	Moment	Group A (n=10)	Group B (n=9)	Total (n=19)
ICP (mmHG)	Baseline	7.26±5.87	7.28±4.2	7.27±5.01
	Post-inflation	23.12±10.86	50.81±27.21	36.24±24.28
	Pre-HS	16.69±6.6	31.96±15.31	23.92±13.69
	Post-HS	15.17±6.26	29.21±16.42	21.82±13.83
	Pre-deflation	16.65±7.96	31.01±19.47	23.45±15.95
	Post-deflation	7.16±6.1	4.69±4.96	5.99±5.58
FVs (cm/s)	Baseline	75.32±48.05	67.93±29.45	71.82±39.43
	Post-inflation	80.32±53.33	59.42±27.84	70.42±43.37
	Pre-HS	89.88±46.06	57.81±26.04	74.69±40.41
	Post-HS	89.17±55.09	79.91±39.05	84.78±47.09
	Pre-deflation	89.76±41.75	71.31±27.27	81.02±35.94
	Post-deflation	94.99±49.08	93.76±35.36	94.41±41.96
FVd (cm/s)	Baseline	39.62±24.53	33.64±12.27	36.79±19.42
	Post-inflation	30.72±16.79	17.69±9.17	24.55±14.93
	Pre-HS	42.23±22.31	21.75±23.08	32.53±24.42
	Post-HS	45.39±24.76	27.55±36.62	36.94±31.41
	Pre-deflation	46.01±20.61	29.58±24.17	38.23±23.3
	Post-deflation	53.14±27.27	48.02±28.17	50.71±27.04
FVm (cm/s)	Baseline	51.52±32.21	45.07±17.72	48.47±25.87
	Post-inflation	47.25±27.97	31.6±14.07	39.84±23.32
	Pre-HS	58.11±28.13	33.77±22.69	46.58±27.94
	Post-HS	59.98±33.89	45±32.02	52.89±33
	Pre-deflation	60.59±26.82	40.45±23.01	51.05±26.49
	Post-deflation	67.09±33.65	63.26±29.07	65.27±30.75
PI	Baseline	0.69±0.15	0.74±0.19	0.71±0.17
	Post-inflation	1.02±0.31	1.3±0.39	1.15±0.37
	Pre-HS	0.82±0.3	2.54±3.68	1.63±2.62
	Post-HS	0.71±0.22	6.59±15.83	3.5±10.98
	Pre-deflation	0.73±0.2	11.73±31.31	5.94±21.62
	Post-deflation	0.63±0.19	0.87±0.65	0.75±0.47

FVs: systolic cerebral blood flow velocity; FVd: diastolic cerebral blood flow velocity; FVm: mean cerebral blood flow velocity; PI: pulsatility index; post-inflation: after balloon inflation; pre- and post-HS: pre- and post-hypertonic solution infusion; pre- and post-deflation: pre- and post-balloon deflation.

Group A: red line; Group B: green line; ICP: intracranial pressure; post-inflation: after balloon inflation; pre- and post-HS: pre- and post-hypertonic solution infusion; pre- and post-deflation: pre- and post-balloon deflation.

**Figure 2.**
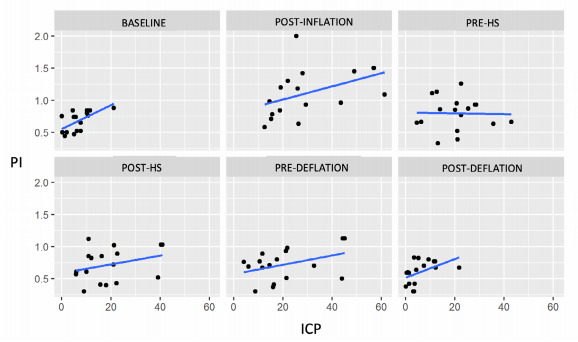
Dispersion between intracranial pressure and pulsatility index, without the animals 15 and 16.

PI: pulsatility index; ICP: intracranial pressure; post-inflation: after balloon inflation; pre- and post-HS: pre- and post-hypertonic solution infusion; pre- and post-deflation: pre- and post-balloon deflation.

From the adjusted model, Table 3 was constructed, comparing the groups for each moment and the differences between groups for each evaluated moment (basal, post-inflation, pre-HS, post-HS, pre-deflation and post-deflation). There was no statistical difference in ICP between groups before and after the end of the experiment. ICP was higher in group B than group A in the moments just after the insufflation, pre-HS, post-HS and pre-deflation. There was no statistical difference between groups in FVs, FVd, FVm, and PI at any point of the experiment. For transcranial Doppler analysis, data of two animals of group B were excluded due to severe hemodynamic instability (Figure 2). Hence, subjects without significant changes in systemic hemodynamics during the procedure were accounted for statistical analysis.

**Table 3.** Multiple comparisons of the intracranial pressure difference between groups B and A at different time points of the experiment.

Multiple comparisons	95%CI	p-value
7-4 mL (Baseline)	0.02 (-11.33-11.37)	0.998
7-4 mL (post-inflation)	27.69 (16.34-39,04)	<0.001
7-4 mL (pre-HS)	15.27 (3.91-26.62)	0.008
7-4 mL (post-HS)	14.04 (2.69-25.39)	0.015
7-4 mL (pre-deflation)	14.36 (3.01-25.71)	0.013
7-4 mL (post-deflation)	-2.47 (-13.82-8.88)	0.670

95%CI: 95% confidence interval; post-inflation: after balloon inflation; pre- and post-HS: pre- and post-hypertonic solution infusion; pre- and post-deflation: pre- and post-balloon deflation.

**Figure 3.**
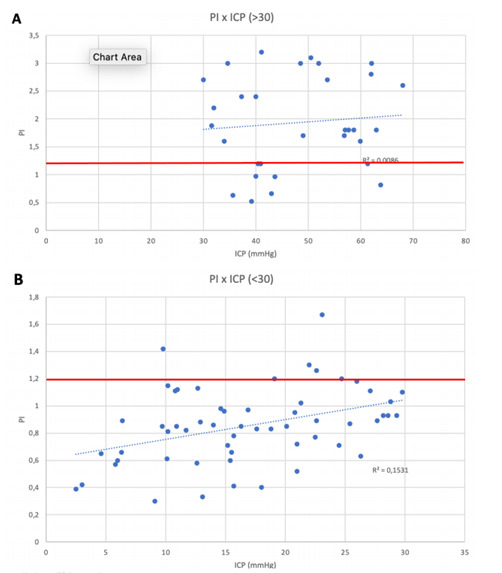
Spearman’s correlation. A positive trend was observed for intracranial pressure and pulsatility index correlation, although a pulsatility indexcut-off value of ≥1.2 (red line) was observed for an intracranial pressure cut-off value of 30 mmHg (A). In our study, animals disclosing intracranial pressure between 20-29 mmHg often presented pulsatility index values under 1.2 (B).

PI: pulsatility index; ICP: intracranial pressure.

There was a moderate correlation between PI and ICP at three moments of the experiment (Spearman correlation coefficients): at baseline (r:0.661), post-inflation (r:0.543), and post-deflation (r:0.578), all with p<0.05. No significant correlation was found between ICP and FVs, FVd, and FVm. The dispersion of the correlations between ICP and PI are presented in Figure 2. It shows that the correlation of ICP with PI at baseline and shortly after balloon inflation is greater than the correlation between ICP and other PI values over time. As the elevation in ICP was varied widely among subjects, a precise cut-off was could not be calculated, although Table 2 indicates that for a sudden severe ICH (group B), PI elevation is progressive and less specific for intervention, except for a rapid relief (balloon deflation could simulate decompressive cranioctomy). Figure 3 shows a positive correlation between PI and ICP, especially with substantial elevation in ICP (>30 mmHg). A moderate elevation in ICP tends to respond better to interventions such as hypertonic saline, and PI will have more negative predictive value in these cases.

## DISCUSSION

The present study makes important contributions in the development of an animal model with induced and reversible ICH. Correlations between ICP- and TCD-derived parameters were calculated. There was a moderate correlation between PI and ICP at three moments of the experiment. From the pre-HS moment to pre-deflation, there was no significant correlation. The variables FVs, FVd, and FVm were not correlated with ICP at any moment. The data obtained are in agreement with those of some studies and in contrast with others, as discussed below.

### Correlation between intracranial pressure invasive monitoring and transcranial Doppler parameters

Invasive ICP monitoring devices have been developed throughout the 20th century and since then have become the gold standard method for this purpose, despite controversial results in some studies^5^ ^,^ ^6^ ^,^ ^19^ ^,^ ^20^ ^,^ ^21^. However, due to its potential complications, such as infection, hemorrhage, and misplacement, numerous studies have been conducted in recent years aimed at developing several non-invasive techniques for estimation of ICP^9^ ^,^ ^22^ ^,^ ^23^ ^,^ ^24^.

TCD is a promising technique with emphasis on PI as a parameter of non-invasive estimation of ICP^9^ ^,^ ^10^ ^,^ ^25^ ^,^ ^26^. TCD is an interesting method because of its availability, portability, and possibility of performing repeated non-invasive tests at bedside.

Bellner et al. investigated the relationship between ICP and TCD PI^9^. They studied 81 patients with various brain lesions (subarachnoid hemorrhage, TBI, and others). It was found a direct relationship between ICP and PI, with a high correlation coefficient (0.938). Similar results were reported in a study of 58 patients with severe TBI treated in the ICU according to the BTF guidelines^27^. Daily TCD was performed for PI comparison with invasive ICP monitoring. There was a strong correlation between PI and ICP, with a 0.779 correlation coefficient in the fifth day.

A retrospective study from Cambridge^10^ included 53 patients who underwent an infusion test (consisting of infusing saline solution in the lumbar space, by lumbar puncture, for the study of circulatory disorders). The values of ICP (measured through the lumbar needle) and TCD blood flow velocities were also measured. One of the parameters studied for noninvasive ICP measurement was the PI obtained with TCD. There was statistical significance in the direct correlation between these two parameters (r: 0.45), showing good potential of PI for noninvasive estimation of ICP.

Other studies have also shown positive results regarding the correlation of ICP with PI in patients with brain lesions^28^ ^,^ ^29^ ^,^ ^30^ ^,^ ^31^. These studies are consistent with the results of our experiment in which a strong correlation was found between PI and ICP at three important moments. The correlation coefficient was 0.543 right after balloon inflation, when ICP elevation occurs. At the other moments, when ICP remains high but stable, PI lost its correlation with ICP. We can infer from this that PI may be important in monitoring the worsening of ICP and may be indicated in patients with this suspicion of deterioration.

Bouzart et al. conducted a prospective multicenter study in France in 2016^25^. A total of 356 patients with mild and moderate TBI (Glasgow between 9 and 15) were studied. Bilateral TCD was performed up to 8 hours post-trauma. The objective was to evaluate TCD as a predictor of secondary neurological deterioration in these patients (which occurred in 6% of study patients) on the seventh day post-trauma. The normal TCD parameter considered was PI less than 1.25 and FVd greater than 25 cm/s. This parameter had sensitivity of 80% and specificity of 79% to predict neurological worsening. The negative predictive value was 98% and the positive predictive value was 18%, suggesting that the normal TCD result is more important in predicting prognosis than the abnormal TCD. Also, this study showed that PI and FVd are related to the prognosis of patients with mild to moderate TBI. This study, despite not evaluating the correlation of the TCD parameters with ICP, reinforces the possible utility of PI in patients with potential risk of neurological worsening.

However, there are many studies in the literature that contradict these positive correlations between ICP and PI. In 2016, another prospective study included 40 TBI patients who were treated in an ICU of a single hospital and who received a parenchymal catheter to monitor ICP^22^. Data were collected from ICP monitoring and TCD. One of the parameters analyzed for non-invasive evaluation of ICP was PI. There was no statistically significant correlation between PI and ICP. The results were consistent with those of Figaji et al., another prospective study that evaluated 34 children with severe TBI, who had ICP monitored^32^. TCD was performed in the middle cerebral artery ipsilateral to the ICP catheter. The aim of this study was to determine the correlation between PI greater than 1 and ICP greater than 20 mmHg, as well as PI less than 1 with ICP less than 20 mmHg. The conclusion of that study was that PI is not a good parameter for noninvasive assessment of ICP in children with TBI. These results are consistent with other published studies^7^ ^,^ ^22^ ^,^ ^23^ ^,^ ^33^ ^,^ ^34^.

### Effect of hypertonic saline solution infusion in the present model

As described in the literature review, hyperosmolar therapy is used to treat cerebral edema and ICH of various etiologies, and mannitol at 20% is the gold standard solution. However, HS, in different concentrations, has been studied for this purpose^35^ ^,^ ^36^. Hypertonic solutions act through the dehydration of brain tissue and decrease the inflammatory response of the brain to injury, as well as causing positive effects on homeostasis and cardiovascular hemodynamics^37^ ^,^ ^38^. Although there was no consensus on the best HS concentration for ICH control, in this study the 3% concentration was used because it was equiosmolar to 20% mannitol. In addition, it has shown good efficacy in intraoperative brain relaxation and in controlling ICH of various causes, with good safety and few side effects^39^ ^,^ ^40^ ^,^ ^41^.

In the current study, groups A and B maintained stable ICP after HS infusion, without the ICP reduction effect demonstrated in other studies. Perhaps the present animal model of ICH is not adequate to evaluate HS effects. Balloon inflation simulates an acute mass effect with a material that does not respond to changes in blood osmolarity. The benefits of HS are postulated as a result of an osmolar effect, which would not affect the balloon. Other effects of HS as increased cardiac output and inhibition of inflammatory changes are also not applicable in this model. This justifies the results obtained in this experiment, in which there was no change in ICP after infusion of HS. Therefore, the ICH model by balloon inflation simulates a disease process that can only be treated by surgical intervention.

Although the present study makes important contributions in the development of an animal model of induced ICH, it has some limitations. First, Doppler evaluations are highly operator-dependent with a significant learning curve. However, only one accurately trained sonographer performed the Doppler exams to minimize this limitation. Second, FVs, FVd, FVm, and PI parameters are also influenced by blood pressure and blood viscosity. Third, the intracranial solution infusions applied in the study were comparable to extremely elevated intracranial mass volume, which is not the most common situation in clinical practice, although suitable for study purposes.

Additionally, the lesions induced in the study were exclusively performed on the frontal lobe of swines. Theoretically, lesions with the same volume in the posterior fossa may disclose a different behavior on blood flow velocities of middle cerebral arteries. Finally, two animals presented hemodynamic instability, refractory to the stabilization attempts made by the researchers, and were excluded from the PI data analysis. Another limitation of this animal model is the absence of blood contact with brain tissue, with absence of inflammatory reactions caused by a true hematoma.

PI is mostly an indicator of cerebral perfusion pressure, as its formula is based on differences between systolic and diastolic velocities. Previous research used 1.4^42^ as the threshold for this index to indicate ICH more accurately. However, in clinical practice, logic leads to individualization, since both intracranial compliance and pressure buffering mechanisms vary from person to person. Rheology, intravascular volume, and the cardiovascular system also play a determining role on cerebral hemodynamics^43^. Thus, the most valuable feature of a non-invasive technique such as TCD may be the opportunity of repeated evaluations and observing particular PI tendency during patient follow-up, associating this with further dynamic variables.

In conclusion, in this experimental study, transcranial Doppler pulsatility index was correlated with ICP monitored by intraparenchymal catheter, especially at the moment of abrupt elevation of ICP. This observation is relevant because similar studies cannot be performed in humans for ethical reasons.

## References

[1] Andrade AF, Paiva WS, Amorim RL, Figueiredo EG, Almeida AN, Brock RS (2011). Continuous ventricular cerebrospinal fluid drainage with intracranial pressure monitoring for management of posttraumatic diffuse brain swelling. Arq Neuro-Psiquiatr.

[2] Bor-Seng-Shu E, Kita WS, Figueiredo EG, Paiva WS, Fonoff ET, Teixeira MJ (2012). Cerebral hemodynamics: concepts of clinical importance. Arq Neuro-Psiquiatr.

[3] Czosnyka M, Hutchinson PJ, Balestreri M, Hiler M, Smielewski P, Pickard JD (2006). Monitoring and interpretation of intracranial pressure after head injury. Acta Neurochir Suppl.

[4] Fukuda T, Hasue M, Ito H (1998). Does traumatic subarachnoid hemorrhage caused by diffuse brain injury cause delayed ischemic brain damage? Comparison with subarachnoid hemorrhage caused by ruptured intracranial aneurysms. Neurosurgery.

[5] Adelson PD, Bratton SL, Carney NA, Chesnut RM, du Coudray HEM, Goldstein B (2003). Guidelines for the acute medical management of severe traumatic brain injury in infants, children, and adolescents. Chapter 7. Intracranial pressure monitoring technology. Pediatr Crit Care Med.

[6] Chesnut RM, Temkin N, Carney N, Dikmen S, Rondina C, Videtta W (2012). A trial of intracranial-pressure monitoring in traumatic brain injury. N Engl J Med.

[7] Zweifel C, Czosnyka M, Carrera E, de Riva N, Pickard JD, Smielewski P (2012). Reliability of the blood flow velocity pulsatility index for assessment of intracranial and cerebral perfusion pressures in head-injured patients. Neurosurgery.

[8] Ragauskas A, Matijosaitis V, Zakelis R, Petrikonis K, Rastenyte D, Piper I (2012). Clinical assessment of noninvasive intracranial pressure absolute value measurement method. Neurology.

[9] Bellner J, Romner B, Reinstrup P, Kristiansson KA, Ryding E, Brandt L (2004). Transcranial Doppler sonography pulsatility index (PI) reflects intracranial pressure (ICP). Surg Neurol.

[10] Cardim D, Czosnyka M, Donnelly J, Robba C, Cabella BCT, Liu X (2016). Assessment of non-invasive ICP during CSF infusion test: an approach with transcranial Doppler. Acta Neurochir (Wien).

[11] Budohoski KP, Schmidt B, Smielewski P, Kasprowicz M, Plontke R, Pickard JD (2012). Non-invasively estimated ICP pulse amplitude strongly correlates with outcome after TBI. Acta Neurochir Suppl.

[12] Robba C, Bacigaluppi S, Cardim D, Donnelly J, Bertuccio A, Czosnyka M (2016). Non-invasive assessment of intracranial pressure. Acta Neurol Scand.

[13] Gregers MCT, Mikkelsen S, Lindvig KP, Brøchner AC (2020). Ketamine as an anesthetic for patients with acute brain injury: a systematic review. Neurocrit Care.

[14] Froese L, Dian J, Batson C, Gomez A, Unger B, Zeiler FA (2020). Cerebrovascular response to propofol, fentanyl, and midazolam in moderate/severe traumatic brain injury: a scoping systematic review of the human and animal literature. Neurotrauma Rep.

[15] Andrade AF, Soares MS, Patriota GC, Belon AR, Paiva WS, Bor-Seng-Shu E (2013). Experimental model of intracranial hypertension with continuous multiparametric monitoring in swine. Arq Neuropsiquiatr.

[16] de Lima Oliveira M, Salinet AM, Nogueira RC, Belon AR, Paiva WS, Jeng BCP (2018). The effects of induction and treatment of intracranial hypertension on cerebral autoregulation: an experimental study. Neurol Res Int.

[17] de-Lima-Oliveira M, Ferreira AA, Belon AR, Salinet AM, Nogueira RC, Ping BC (2020). The influence of intracranial hypertension on static cerebral autoregulation. Brain Inj.

[18] Drikvandi R (2020). Nonlinear mixed-effects models with misspecified random-effects distribution. Pharm Stat.

[19] Cremer OL, van Dijk GW, van Wensen E, Brekelmans GJF, Moons KGMM, Leenen LPHL (2005). Effect of intracranial pressure monitoring and targeted intensive care on functional outcome after severe head injury. Crit Care Med.

[20] Aiolfi A, Benjamin E, Khor D, Inaba K, Lam L, Demetriades D (2017). Brain trauma foundation guidelines for intracranial pressure monitoring: compliance and effect on outcome. World J Surg.

[21] Le Roux P, Menon DK, Citerio G, Vespa P, Bader MK, Brophy G (2014). The International Multidisciplinary Consensus Conference on Multimodality Monitoring in Neurocritical Care: evidentiary tables: a statement for healthcare professionals from the Neurocritical Care Society and the European Society of Intensive Care Medicine. Neurocrit Care.

[22] Cardim D, Robba C, Donnelly J, Bohdanowicz M, Schmidt B, Damian M (2016). Prospective study on noninvasive assessment of intracranial pressure in traumatic brain-injured patients: comparison of four methods. J Neurotrauma.

[23] Robba C, Cardim D, Tajsic T, Pietersen J, Bulman M, Donnelly J (2017). Ultrasound non-invasive measurement of intracranial pressure in neurointensive care: A prospective observational study. PLoS Med.

[24] Roh D, Park S (2016). Brain multimodality monitoring: updated perspectives. Curr Neurol Neurosci Rep.

[25] Bouzat P, Almeras L, Manhes P, Sanders L, Levrat A, David JS (2016). Transcranial Doppler to predict neurologic outcome after mild to moderate traumatic brain injury. Anesthesiology.

[26] Cardim D, Robba C, Bohdanowicz M, Donnelly J, Cabella B, Liu X (2016). Non-invasive monitoring of intracranial pressure using transcranial doppler ultrasonography: is it possible?. Neurocrit Care.

[27] Gura M, Elmaci I, Sari R, Coskun N (2011). Correlation of pulsatility index with intracranial pressure in traumatic brain injury. Turk Neurosurg.

[28] Czosnyka M, Matta BF, Smielewski P, Kirkpatrick PJ, Pickard JD (1998). Cerebral perfusion pressure in head-injured patients: a noninvasive assessment using transcranial Doppler ultrasonography. J Neurosurg.

[29] Melo JR, Di Rocco F, Blanot S, Cuttaree H, Sainte-Rose C, Oliveira-Filho J (2011). Transcranial Doppler can predict intracranial hypertension in children with severe traumatic brain injuries. Childs Nerv Syst.

[30] O’Brien NF, Maa T, Reuter-Rice K (2015). Noninvasive screening for intracranial hypertension in children with acute, severe traumatic brain injury. J Neurosurg Pediatr.

[31] Wang Y, Duan YY, Zhou HY, Yuan LJ, Zhang L, Wang W (2014). Middle cerebral arterial flow changes on transcranial color and spectral Doppler sonography in patients with increased intracranial pressure. J Ultrasound Med.

[32] Figaji AA, Zwane E, Fieggen AG, Siesjo P, Peter JC (2009). Transcranial Doppler pulsatility index is not a reliable indicator of intracranial pressure in children with severe traumatic brain injury. Surg Neurol.

[33] Hanlo PW, Gooskens RH, Nijhuis IJ, Faber JA, Peters RJ, van Huffelen AC (1995). Value of transcranial Doppler indices in predicting raised ICP in infantile hydrocephalus. A study with review of the literature. Childs Nerv Syst.

[34] Morgalla MH, Magunia H (2016). Noninvasive measurement of intracranial pressure via the pulsatility index on transcranial Doppler sonography: Is improvement possible?. J Clin Ultrasound.

[35] Diringer MN (2013). New trends in hyperosmolar therapy?. Curr Opin Crit Care.

[36] Stocchetti N, Maas AI (2014). Traumatic intracranial hypertension. N Engl J Med.

[37] Huang X, Yang L, Ye J, He S, Wang B (2020). Equimolar doses of hypertonic agents (saline or mannitol) in the treatment of intracranial hypertension after severe traumatic brain injury. Medicine (Baltimore).

[38] Suarez JI (2004). Hypertonic saline for cerebral edema and elevated intracranial pressure. Cleve Clin J Med.

[39] Khanna S, Davis D, Peterson B, Fisher B, Tung H, O’Quigley J (2000). Use of hypertonic saline in the treatment of severe refractory posttraumatic intracranial hypertension in pediatric traumatic brain injury. Crit Care Med.

[40] Prabhakar H, Singh GP, Anand V, Kalaivani M (2014). Mannitol versus hypertonic saline for brain relaxation in patients undergoing craniotomy. Cochrane Database Syst Rev.

[41] Sokhal N, Rath GP, Chaturvedi A, Singh M, Dash HH (2017). Comparison of 20% mannitol and 3% hypertonic saline on intracranial pressure and systemic hemodynamics. J Clin Neurosci.

[42] Robba C, Pozzebon S, Moro B, Vincent JL, Creteur J, Taccone FS (2020). Multimodal non-invasive assessment of intracranial hypertension: an observational study. Crit Care.

[43] Alexandrov AV, Sloan MA, Wong LK, Douville C, Razumovsky AY, Koroshetz WJ (2007). Practice standards for transcranial Doppler ultrasound: part I--test performance. J Neuroimaging.

